# Supplementary material for: Splice-Junction-Based Mapping of Alternative Isoforms in the Human Proteome
Source: Cell Rep. Author manuscript; Available in PMC 2020 Jan 15. (PMC6961840; doi:10.1016/j.celrep.2019.11.026)
Supplement: 2 [file NIHMS1546469-supplement-2.zip › DF1/PXD010154/Liver/Liver_54_PPP1R35_AEEQPGLGPGAAGR.pdf]

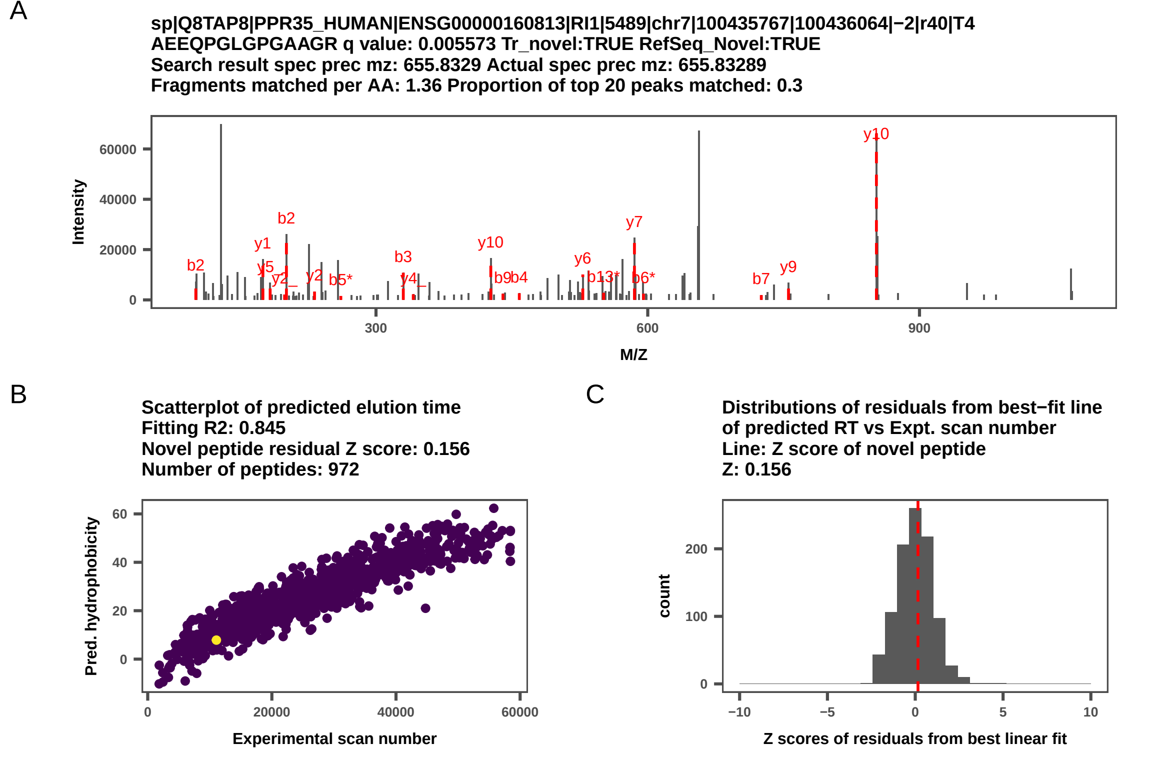
A
sp|Q8TAP8|PPR35_HUMAN|ENSG00000160813|RI1|5489|chr7|100435767|100436064|−2|r40|T4
AEEQPGLGPGAAGR q value: 0.005573 Tr_novel:TRUE RefSeq_Novel:TRUE
Search result spec prec mz: 655.8329 Actual spec prec mz: 655.83289
Fragments matched per AA: 1.36 Proportion of top 20 peaks matched: 0.3
Intensity
60000
40000
20000
0
b2
y1
y5
y2_
b2
y2
b5*
b3
y4_
y10
b9
b4
y6
b13*
y7
b6*
b7
y9
y10
300
600
900
M/Z
B
Scatterplot of predicted elution time
Fitting R2: 0.845
Novel peptide residual Z score: 0.156
Number of peptides: 972
Pred. hydrophobicity
60
40
20
0
0
20000
40000
60000
Experimental scan number
C
Distributions of residuals from best−fit line
of predicted RT vs Expt. scan number
Line: Z score of novel peptide
Z: 0.156
count
200
100
0
−10
−5
0
5
10
Z scores of residuals from best linear fit
